# Supplementary material for: Efficacy of Autologous Intrauterine Infusion of Platelet-Rich Plasma in Patients with Unexplained Repeated Implantation Failures in Embryo Transfer: A Systematic Review and Meta-Analysis
Source: J Clin Med. 2022 Nov 15;11(22):6753. doi: 10.3390/jcm11226753 (PMC9697934; doi:10.3390/jcm11226753)
Supplement: Supplementary file 1 [file jcm-11-06753-s001.zip › Supplementary Figure.pdf]

Figure S1: (a) Risk of bias graph and (b) risk of bias summary

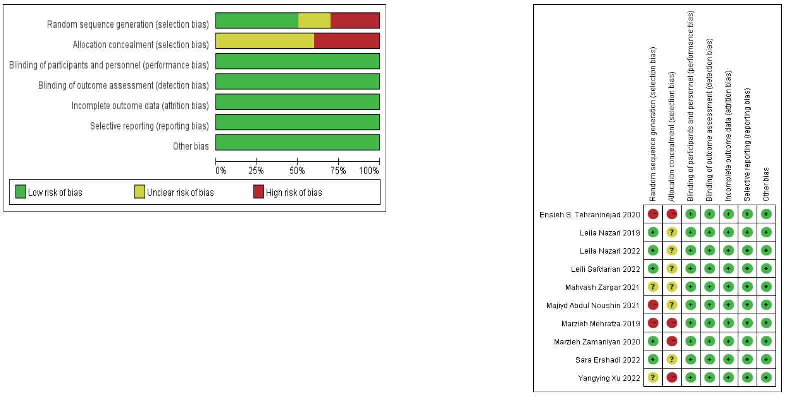

(a) (b)

Figure S2: Funnel plot of meta-analysis of 10 included studies.

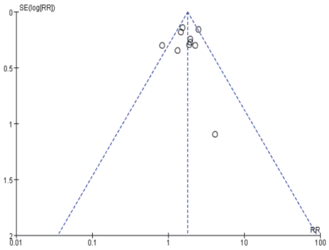

Figure S3: Funnel plot of meta-analysis of 10 included studies excluding PRP dosage.

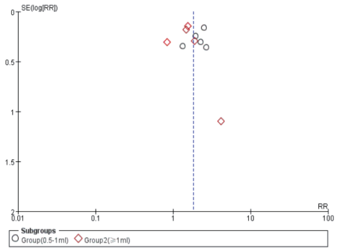

Figure S4: Funnel plot of meta-analysis of 10 included studies excluding study design.

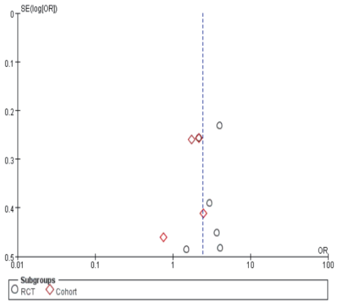

Figure S5: Forest plot of RR, 95% CI, and heterogeneity in studies that evaluated the risk of live birth rates in interventions versus controls

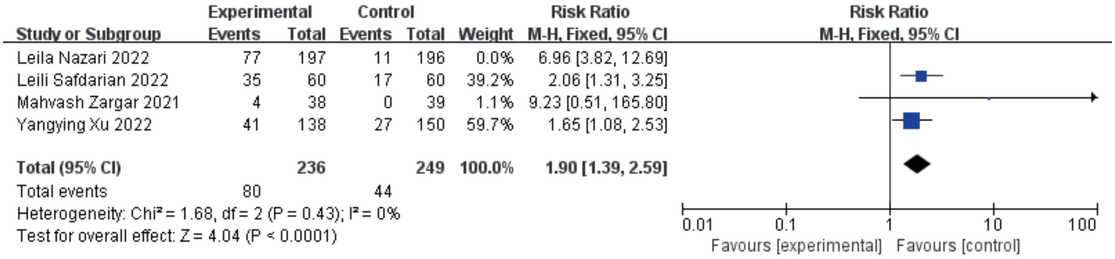

Study or Subgroup	Experimental Events	Experimental Total	Control Events	Control Total	Weight	Risk Ratio M-H, Fixed, 95% CI
Leila Nazari 2022	77	197	11	196	0.0%	6.96 [3.82, 12.69]
Leili Safdarian 2022	35	60	17	60	39.2%	2.06 [1.31, 3.25]
Mahvash Zargar 2021	4	38	0	39	1.1%	9.23 [0.51, 165.80]
Yangying Xu 2022	41	138	27	150	59.7%	1.65 [1.08, 2.53]
**Total (95% CI)**		**236**		**249**	**100.0%**	**1.90 [1.39, 2.59]**
Total events	80		44			

Heterogeneity: Chi² = 1.68, df = 2 (P = 0.43); I² = 0%
Test for overall effect: Z = 4.04 (P < 0.0001)
