# Supplementary material for: Efficacy of Autologous Intrauterine Infusion of Platelet-Rich Plasma in Patients with Unexplained Repeated Implantation Failures in Embryo Transfer: A Systematic Review and Meta-Analysis
Source: J Clin Med. 2022 Nov 15;11(22):6753. doi: 10.3390/jcm11226753 (PMC9697934; doi:10.3390/jcm11226753)
Supplement: Supplementary file 1 [file jcm-11-06753-s001.zip › Table S1.pdf]

Endometrial preparation	Transfer type	Outcome measures	Endometrial thickness(mm)		Time of embryo transfer	
			Control	Case	Control	Case
Hormone replacement treatment protocol and natural cycle	Frozen embryo transfer	The rates of live birth, clinical pregnancy, positive β-HCG, miscarriage and implantation	10.06 ± 2.16	9.80 ± 2.27	Cleavage stage or blastocyst stage	Cleavage stage or blastocyst stage
	Frozen embryo transfer	The rates of clinical pregnancy, implantation, ongoing pregnancy, live birth,miscarriage, multiple pregnancy and preterm delivery	＞7	＞7	Blastocyst stage	Blastocyst stage
Hormone replacement therapy	Frozen embryo transfer	The rates of clinical pregnancy, multiple pregnancy and live birth	8.9 ± 4.9	9.2 ± 3.2	Blastocyst stage	Blastocyst stage
Hormone replacement therapy	Fresh embryo transfer and frozen embryo transfer	The rates of fertility, embryo implantation, and pregnancy maintenance	＞7	＞7	Cleavage stage	Cleavage stage
Long GnRH agonist, GnRH antagonist	Frozen embryo transfer	The rates of clinical pregnancy, multiple pregnancy, miscarriage, ongoing pregnancy and implantation	10.00 ± 0.93, ＞7	13.15 ± 1.42, ＞7	Blastocyst stage	Blastocyst stage
Hormone replacement therapy	Frozen embryo transfer	The rates of clinical pregnancy, and ongoing pregnancy	≥7	≥7	Blastocyst stage	Blastocyst stage
Hormone replacement therapy	Frozen embryo transfer	The rate of ongoing pregnancy, live birth, clinical pregnancy, and miscarriage	9.64 ±0.66, ≥8	9.31±1.17, ≥8	Cleavage stage or blastocyst stage	Cleavage stage or blastocyst stage
Hormone replacement therapy	Frozen embryo transfer	The rates of clinical prgnancy	≥7	≥7	Blastocyst stage	Blastocyst stage
Not mentioned	Fresh embryo transfer and frozen embryo transfer	The rates of chemical pregnancy, clinical pregnancy and abortion	9.5±1.20	9.12±1.29	Cleavage stage	Cleavage stage
Not mentioned	Fresh embryo transfer and frozen embryo transfer	The rates of implantation, and clinical pregnancy	≥7	≥7	Cleavage stage or blastocyst stage	Cleavage stage or blastocyst stage

Sample size		Intervention(s)	Control	Time of PRP infusion
Control	Case			
150	138	Intrauterine infusion of 1 ml PRP	Underwent ET without intrauterine infusion of PRP	48–72 hours before ET
60	60	Intrauterine infusion of 0.5 ml PRP	Underwent ET without intrauterine infusion of PRP	48 hours before ET
196	197	Intrauterine infusion of 0.5 ml PRP	Underwent ET without intrauterine infusion of PRP	48 hours before ET
39	38	Intrauterine infusion of 1.5 ml PRP	Underwent ET without intrauterine infusion of PRP	48 hours before ET
43	55	Intrauterine infusion of 0.5 ml	Underwent ET without intrauterine infusion of PRP	48 hours before ET
43	42	Intrauterine infusion of 1 ml PRP	Underwent ET without intrauterine infusion of PRP	2 days before ET
154	109	treatment with PRP intrauterine PRP(1 ml)	Underwent ET without intrauterine infusion of PRP	Intrauterine PRP during the index frozen embryo transfer preparation cycle
48	49	Intrauterine infusion of 0.5 ml PRP	Underwent ET without intrauterine infusion of PRP	48 hours before ET
45	40	Intrauterine infusion of 0.5 ml PRP	Underwent ET without intrauterine infusion of PRP	48 hours before ET
56	67	Intrauterine infusion of 1 ml lympho-PRP	A single administration of 300ug recombinant GCSF and two hours before ET	2 days before ET

Population	Exclusion criteria
Patients aged 23 to 40 years who had three or more consecutive failed embryo implantations with good-quality embryos(at least 6 cleavage-stage embryos or three blastocysts)	Abnormal karyotype from either partner, uterine defects evidence, ultrasonographic evidence of hydrosalpinx, infections, endocrine problems, coagulation defects or autoimmune defects
Patients aged 23 to 40 years who failed to conceive after three or more ET with high-quality embryos and had at least one frozen good-quality blastocyst-stage embryo	Chromosomal and genetic disorders, hematological and immunological disorders, hormonal disorders, uterine abnormality(congenital or acquired), body mass index above 30 kg/m2, severe endometriosis, and patients with cancellation history of the previous ET due to a thin endometrium(≤7 mm) in hormone
Patients aged 18 to 38 years who failed to achieve pregnancy after three or more embryo transfers with high-quality embryos	Immunological abnormalities, inflammatory conditions, hormonal or anatomical disorders, PCOS, OHSS, endometriosis, presence of space-occupying lesions, history of miscarriage or ectopic pregnancy, myomas, polyps, adhesions, previous pelvic surgeries, failed fertilization, and less than two embryos available for transfer, participants with a severe male factor of their spouses and chromosomal abnormalities
Patients below 41 years with at least two IVF failures	Chromosomal, genetic, and uterine abnormalities, hematological or immunological disorders, hormonal disorders, and embryos that arise from such maternal and paternal abnormalities
Patients between 20–40 years who failed to be pregnant after three or more good quality embryo transfer of embryos with good quality	Hematologic disorders, immunologic disorders, hormonal disorders, chromosomal and genetic anomalies, and renal failure
Patients below 35 years who failed to transfer at least four good-quality embryos in at least three fresh or frozen cycles	Age ≥35 years, endometrial thickness <7 mm, basal follicle-stimulating hormone levels >10 mIU/mL, severe male factors such as azoospermia, intrauterine disorders, thrombophilia, thyroid dysfunction, positive antiphospholipid antibodies or chromosomal abnormality in a couple
Patients aged below 40 years with a history of RIF(failed to achieve a clinical pregnancy after ≥3 ET cycles with at least a total of four good quality cleavage/blastocyst-stage embryos derived from autologous gametes transferred in endometrium of ≥8mm thickness) undergoing frozen embryo transfer	BMI≥30 kg/m2, congenital and untreated acquired uterine abnormalities, untreated hydrosalpinges, poor ovarian responder as per the Bologna criteria, thrombophilia, or uncontrolled endocrine or hematologic dysfunction; those undergoing preimplantation genetic testing cycles; and those who had thin endometrium(<8 mm) in the index frozen embryo transfer cycle, severe male factor infertility, difficult ET, only poor-quality embryos available, and couple with genetic and chromosomal abnormalities
Patients aged below 40 years with a history of RIF who failed to conceive after 3 or more embryo transfers with high-quality embryos and candidates for frozen embryo transfer	Uterine abnormalities, hormonal disorders, immunological and haematological disorders, azoospermia, testicular sperm extraction or aspiration, anatomical disorders of the male genital tract, varicocele and chromosomal abnormalities in the couples
Patients aged below 40 years with a history of a history of two to three IVF failures	Any uterine anomalies, an underlying disease, taking any specific medication, no proper frozen embryo transferus for transfer on the day of ET, insufficient endometrial thickness for ET, and reluctance toward participation
Patients aged 18-40 years with a history of more than 2 repeated failed ET	Not mentioned

Table S1. Characteristics of the included studies

Study(Author name & Publication)	Country	Study design
Yangying Xu 2022	China	Cohort
Leili Safdarian 2022	Iran	RCT
Leila Nazari 2022	Iran	RCT
Mahvash Zargar 2021	Iran	RCT
Marzieh Zamaniyan 2020	Iran	RCT
Ensieh S. Tehraninejad 2020	Iran	Cohort
Majiyd Abdul Noushin 2021	India	Cohort
Leila Nazari 2019	Iran	RCT
Sara Ershadi 2022	Iran	RCT
Marzieh Mehrafza 2019	Iran	Cohort
