# Supplementary material for: Efficacy of Autologous Intrauterine Infusion of Platelet-Rich Plasma in Patients with Unexplained Repeated Implantation Failures in Embryo Transfer: A Systematic Review and Meta-Analysis
Source: J Clin Med. 2022 Nov 15;11(22):6753. doi: 10.3390/jcm11226753 (PMC9697934; doi:10.3390/jcm11226753)
Supplement: Supplementary file 1 [file jcm-11-06753-s001.zip › Table S2.pdf]

Table S2. Reasons of excluded studies

Study	Excluded reasons
Tina Tu-Thu Ngoc Nguyen 2022	Animal experiments or cell experiments
Min Kyoung Kim 2022	Animal experiments or cell experiments
Ryan M Brown 2022	Animal experiments or cell experiments
Scarlet Babian 2022	Animal experiments or cell experiments
T. T. T. N. Nguyen 2022	Animal experiments or cell experiments
M. K. Kim 2021	Animal experiments or cell experiments
Konstantinos Sfakianoudis 2019	Case reports
T. T. T. N. Nguyen 2019	Animal experiments or cell experiments
Natalia Juliana Nardelli Gonçalves 2020	Animal experiments or cell experiments
Marzie Farimani 2017	Case reports
S. J. Russell 2022	Self pro-post study
Afsaneh Shah Bakhsh 2022	Poor-quality study
Fateme Aghajanzadeh 2020	Self pro-post study
Yihsien Enatsu 2021	Self pro-post study
